# Supplementary material for: The Spatial Diffusion of Cherry Leaf Roll Virus Revealed by a Bayesian Phylodynamic Analysis
Source: Viruses. 2022 Oct 1;14(10):2179. doi: 10.3390/v14102179 (PMC9612246; doi:10.3390/v14102179)
Supplement: Supplementary file 1 [file viruses-14-02179-s001.zip › Table S1.pdf]

Table S1 Cherry leaf roll virus isolates used in this study

No.	Isolate	Country	Collection date	Accession No.
1	Bpub5	Finland: Rovaniemi, urban park	2011	LN714385
2	Bpub20a	Finland: Rovaniemi, urban park	2011	LN714386
3	Bpub320b	Finland: Rovaniemi, urban park	2011	LN714387
4	Bpen18a	Finland: Rovaniemi, urban park	2011	LN714388
5	Bpub19	Finland: Rovaniemi, urban park	2011	LN714389
6	Bpub362a	Finland: Rovaniemi, urban park	2011	LN714390
7	Bpub401	Finland: Rovaniemi, urban park	2011	LN714391
8	Bpub 20B	Finland: Rovaniemi, HU Berlin-grafted	2011	LN714392
9	Bpub 320A	Finland: Rovaniemi, HU Berlin-grafted	2011	LN714393
10	Bpub13b	Finland: Rovaniemi, urban park	2011	LN714394
11	Bpub 3B	Finland: Rovaniemi, HU Berlin-grafted	2011	LN714395
12	Bpub 7B	Finland: Rovaniemi, HU Berlin-grafted	2011	LN714396
13	Bpub3	Finland: Rovaniemi, urban park	2011	LN714397
14	Bpub 4A	Finland: Rovaniemi, HU Berlin-grafted	2011	LN714398
15	Bpub 7Eb	Finland: Rovaniemi, HU Berlin-grafted	2011	LN714399
16	Bpub 7D	Finland: Rovaniemi, HU Berlin-grafted	2011	LN714400
17	Bpub 3D	Finland: Rovaniemi, HU Berlin-grafted	2011	LN714402
18	Bpub 3A	Finland: Rovaniemi, HU Berlin-grafted	2011	LN714403
19	Bpub 3L	Finland: Rovaniemi, HU Berlin-grafted	2011	LN714404
20	Bpub 3C	Finland: Rovaniemi, HU Berlin-grafted	2011	LN714405
21	Bpub13a	Finland: Rovaniemi, urban park	2011	LN714406
22	Bpub4c	Finland: Rovaniemi, urban park	2011	LN714412
23	Bpub362b	Finland: Rovaniemi, urban park	2011	LN714413
24	Bpub7a	Finland: Rovaniemi, urban park	2011	LN714414
25	Bpub12	Finland: Rovaniemi, urban park	2011	LN714415
26	Bpub 320B	Finland: Rovaniemi, HU Berlin-grafted	2011	LN714416
27	Bpub 4D	Finland: Rovaniemi, HU Berlin-grafted	2011	LN714417
28	Bpub4b	Finland: Rovaniemi, urban park	2011	LN714418
29	Bpen18b	Finland: Rovaniemi, urban park	2011	LN714420
30	Bpub320a	Finland: Rovaniemi, urban park	2011	LN714421
31	Bpub20b	Finland: Rovaniemi, urban park	2011	LN714422
32	Bpub4a	Finland: Rovaniemi, urban park	2011	LN714423
33	Bpub7b	Finland: Rovaniemi, urban park	2011	LN714424
34	Bpub6	Finland: Rovaniemi, urban park	2011	LN714426
35	Bpub14	Finland: Rovaniemi, urban park	2011	LN714427
36	E648	France	1990	KF779176
37	Cors1	France: Corsica, Col de Vergio	2014	LN864972
38	Cors2	France: Corsica, Col de Vergio	2014	LN864973
39	Cors3	France: Corsica, Col de Vergio	2014	LN864974
40	Cors4	France: Corsica, Col de Vergio	2014	LN864975
41	Cors5	France: Corsica, Col de Vergio	2014	LN864976

42	Cors7	France: Corsica, Col de Vergio	2014	LN864977
43	Cors8	France: Corsica, Col de Vergio	2014	LN864978
44	Cors9	France: Corsica, Col de Vergio	2014	LN864979
45	Cors11	France: Corsica, Col de Vergio	2014	LN864980
46	E395	Germany: Bornheim	1987	FR851462
47	PV0278	Germany: Fuchseck	1990	KF779172
48	PV0276	Germany: Lowenburger Hof	2000	KF779173
49	E120	Germany: Berlin-Spandau	2001	KF779174
50	E326	Germany: Bonn-Oberkassel	1990	KF779175
51	E327	Germany: Bonn	1990	KF779180
52	E441	Germany: Aschersleben	2002	KF779181
53	E603	Germany: Werder	2002	KF779182
54	E693	Germany: Titisee-Neustadt	2000	KF779184
55	Bpen 5Aa	Germany: Berlin, HU Berlin-grafted	2011	LN714407
56	Bpen 5Ab	Germany: Berlin, HU Berlin-grafted	2011	LN714408
57	Bpen 5Ba	Germany: Berlin, HU Berlin-grafted	2011	LN714409
58	Bpen 5Bb	Germany: Berlin, HU Berlin-grafted	2011	LN714410
59	Bpen 5Bc	Germany: Berlin, HU Berlin-grafted	2011	LN714411
60	Bpen5	Germany: Berlin, urban park	2011	LN714419
61	Bpen 5C	Germany: Berlin, HU Berlin-grafted	2011	LN714425
62	E327	Germany: Bonn	1990	LT883166
63	E51448	Germany: Berlin	2010	LT883168
64	739	New Zealand	2011	KC937026
65	54	New Zealand	2011	KC937027
66	FNW	New Zealand	2011	KC937028
67	1978	New Zealand	2011	KC937029
68	737	New Zealand	1980	KC937030
69	441	New Zealand	1980	KC937031
70	Actindia-1	New Zealand: Auckland	2011	KF779162
71	Actindia-2	New Zealand	2007	KF779163
72	Actindia-3	New Zealand: Te Puke	2002	KF779164
73	Malus	New Zealand: Waikato	2011	KF779166
74	Plantago	New Zealand: Auckland	2012	KF779167
75	Ribes	New Zealand: Hawkes Bay	2007	KF779168
76	Rubus	New Zealand	1978	KF779169
77	Rumex-1	New Zealand: Auckland	2005	KF779170
78	Rumex-2	New Zealand: Auckland	2011	KF779171
79	Olm1	USA	2011	JN104385
80	W8	USA: California	1980	KF779179
81	E804	USA	1967	KF779183
