# Supplementary material for: The Spatial Diffusion of Cherry Leaf Roll Virus Revealed by a Bayesian Phylodynamic Analysis
Source: Viruses. 2022 Oct 1;14(10):2179. doi: 10.3390/v14102179 (PMC9612246; doi:10.3390/v14102179)
Supplement: Supplementary file 1 [file viruses-14-02179-s001.zip › Table S2.pdf]

Table S2 Marginal likelihoods of different combinations of clock model and tree prior

Model of rate variation	Coalescent tree prior	Path sampling	Stepping-stone sampling
Strict clock	Bayesian skyline	-5846.808	-5843.341
Strict clock	Exponential growth	-5848.570	-5846.195
Strict clock	Constant size	-5846.377	-5845.583
Uncorrelated lognormal relaxed clock	Bayesian skyline	-5839.823	-5842.351
Uncorrelated lognormal relaxed clock	Exponential growth	-5848.108	-5842.407
**Uncorrelated lognormal relaxed clock**	**Constant size**	**-5836.270**	**-5839.145**

The best-fitting tree prior and molecular clock model are indicated in bold font.
