# Supplementary material for: The Spatial Diffusion of Cherry Leaf Roll Virus Revealed by a Bayesian Phylodynamic Analysis
Source: Viruses. 2022 Oct 1;14(10):2179. doi: 10.3390/v14102179 (PMC9612246; doi:10.3390/v14102179)
Supplement: Supplementary file 1 [file viruses-14-02179-s001.zip › Table S3.pdf]

Table S3 Statistically supported migration rates of cherry leaf roll virus estimated from the coat protein sequence

From	To	Mean migration rate	Indicator [a]	Bayes factor [b]
Finland	Germany	1.96	1.00	2275.41
Germany	Finland	1.07	1.00	2022.34
Germany	France	1.14	0.80	8.98
New Zealand	Germany	0.82	0.67	4.49

[a] Posterior probability of observing a non-zero migration rate in the sampled trees.

[b] Only statistically supported migrations with indicator values >0. 50 and BF >3 are shown.

n/a, no data available.
