# Supplementary figures and images for: The Spatial Diffusion of Cherry Leaf Roll Virus Revealed by a Bayesian Phylodynamic Analysis
Source: Viruses. 2022 Oct 1;14(10):2179. doi: 10.3390/v14102179 (PMC9612246; doi:10.3390/v14102179)

**Figure S1**

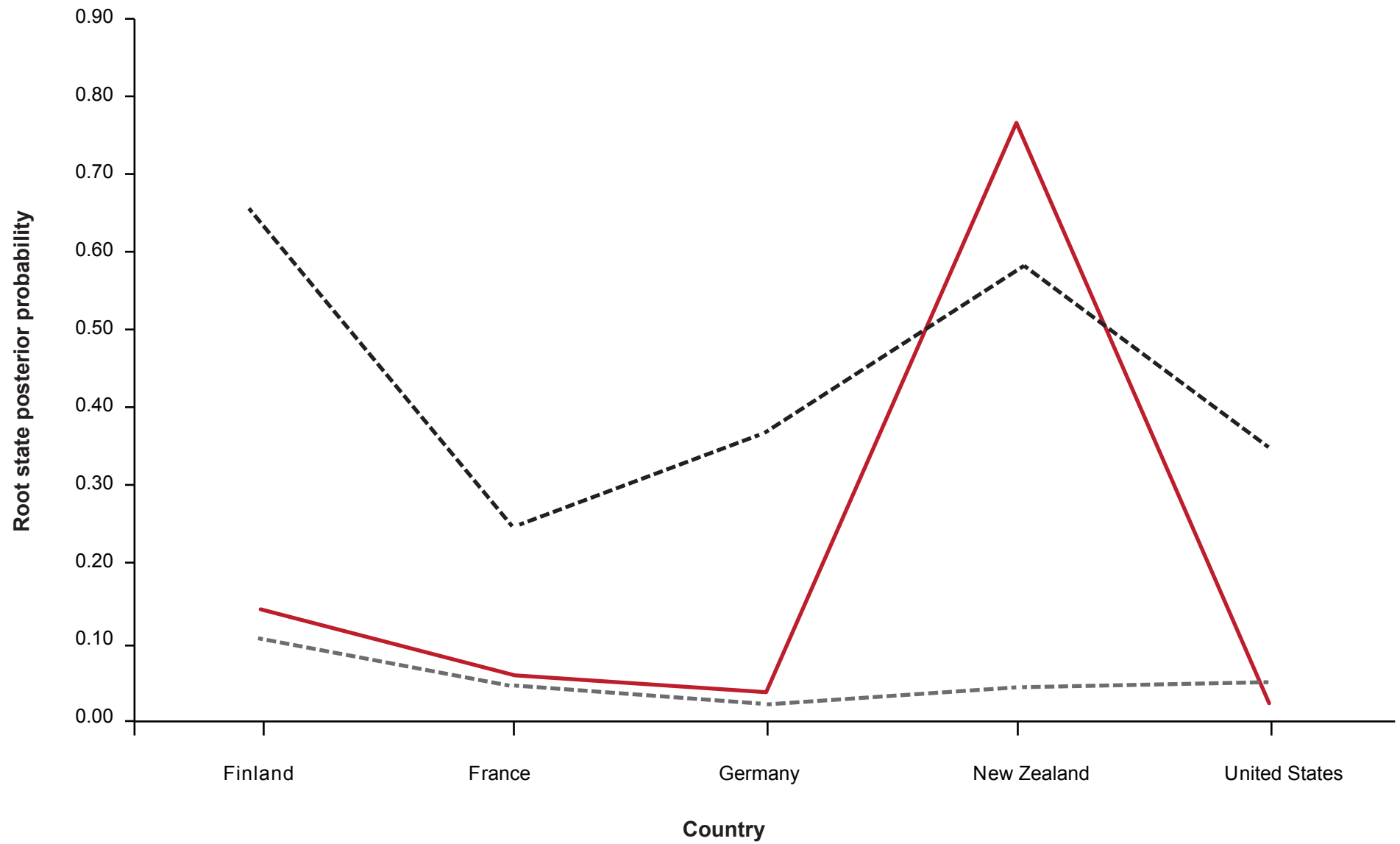

Supplement: Supplementary file 1 [file viruses-14-02179-s001.zip › Figure S1.pdf]

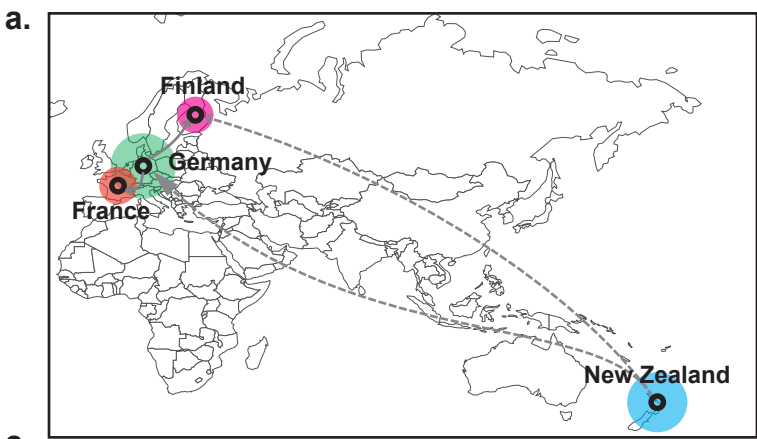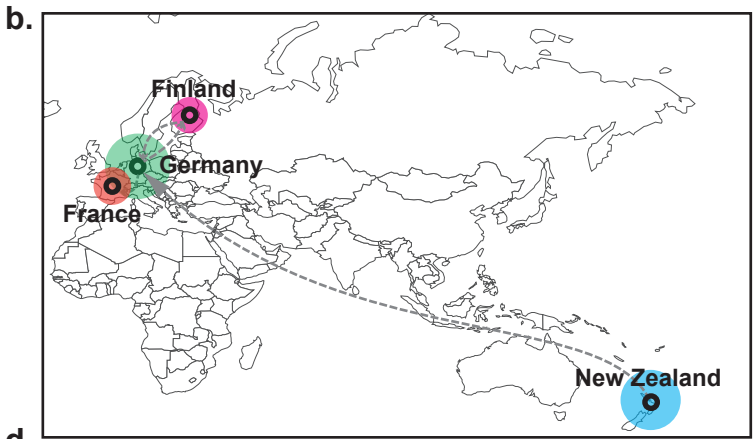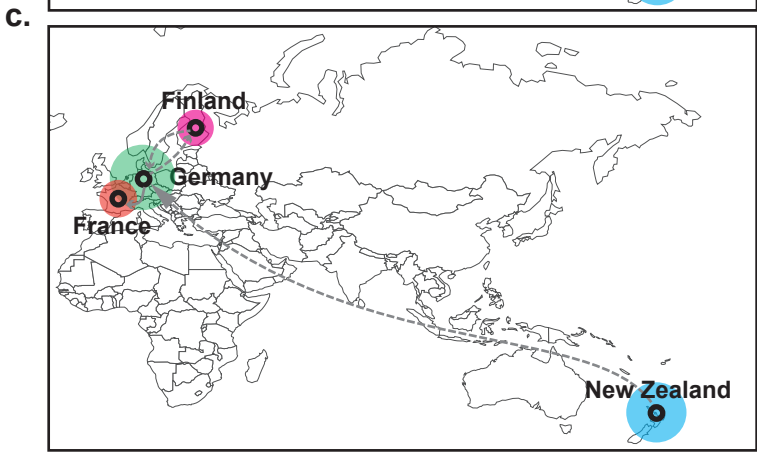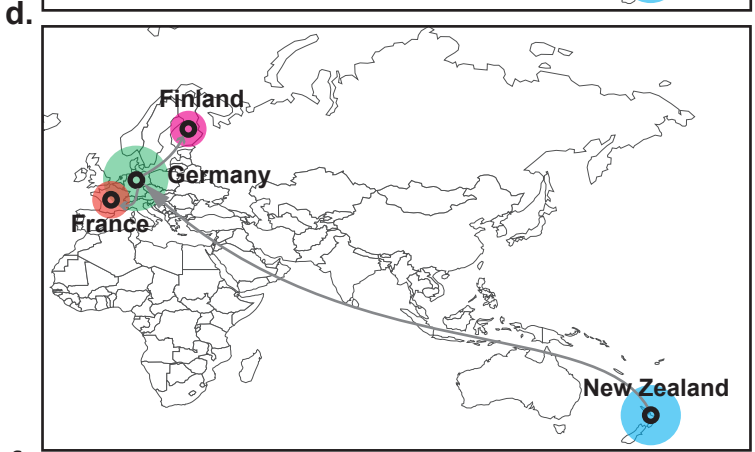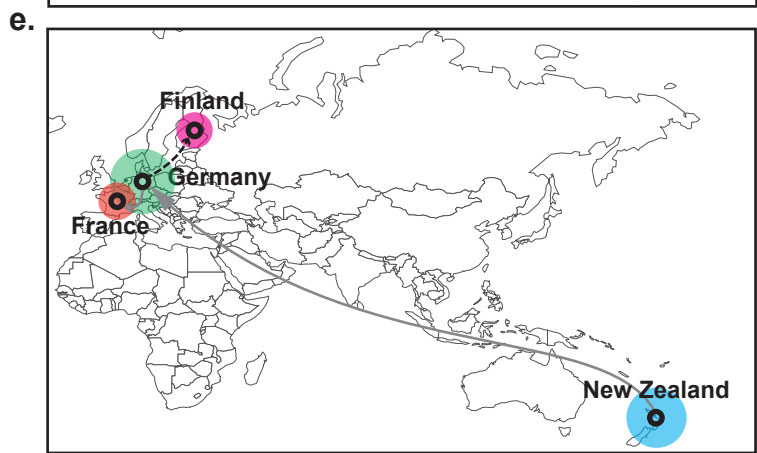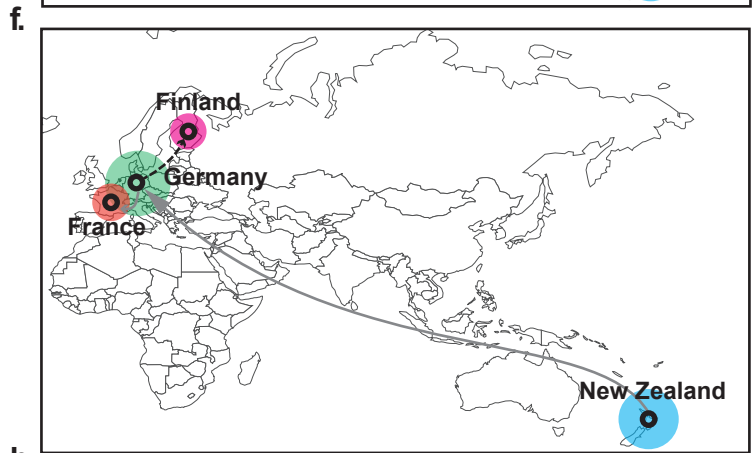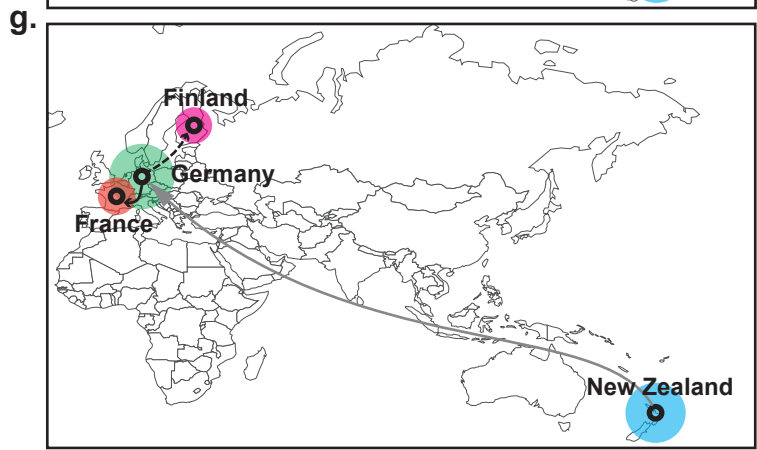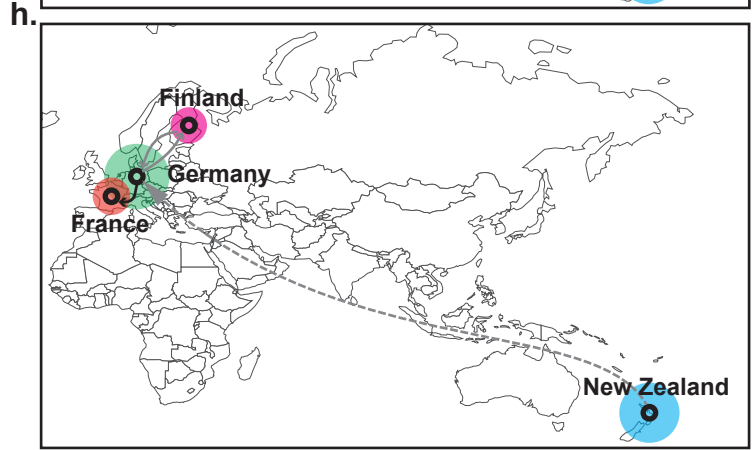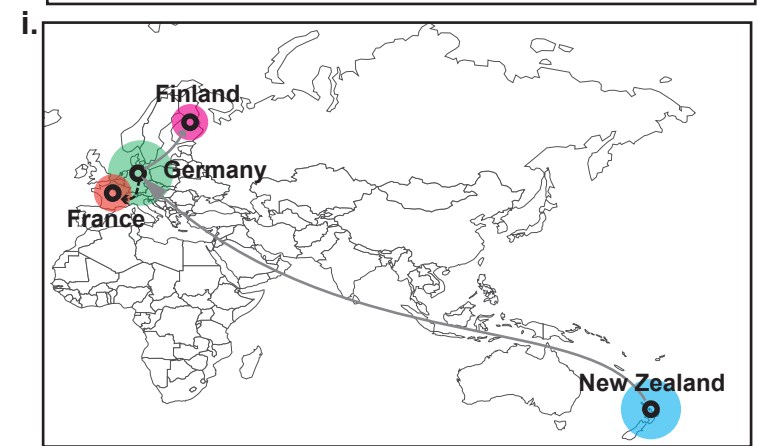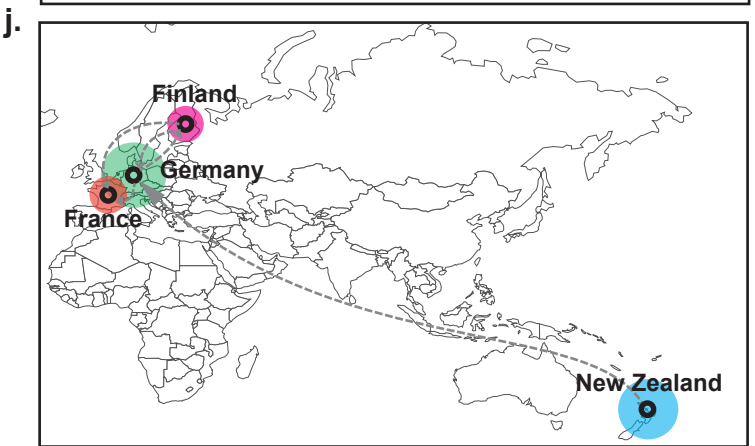

Supplement: Supplementary file 1 [file viruses-14-02179-s001.zip › Figure S2.pdf]
